# Regulation of osteoclastogenesis by mast cell in rheumatoid arthritis

**DOI:** 10.1186/s13075-021-02491-1

**Published:** 2021-04-21

**Authors:** Kyoung-Woon Kim, Bo-Mi Kim, Ji-Yeon Won, Hong-Ki Min, Kyung-Ann Lee, Sang-Heon Lee, Hae-Rim Kim

**Affiliations:** 1 R&D Center, OncoInsight Co. Ltd, Seoul, South Korea; 2 Laboratory of Stem Cell, NEXEL, Seoul, South Korea; 3 Division of Rheumatology, Department of Internal Medicine, Research Institute of Medical Science, Konkuk University School of Medicine, 120-1 Neungdong-ro, Gwangjin-gu, Seoul, 05030 South Korea; 4 Division of Rheumatology, Department of Internal Medicine, Soonchunhyang University College of Medicine, Seoul, South Korea

**Keywords:** Rheumatoid arthritis, Mast cell, Osteoclast, Cytokine

## Abstract

**Background:**

In the pathogenesis of rheumatoid arthritis (RA), the role of mast cells has not been revealed clearly. We aimed to define the inflammatory and tissue-destructive roles of mast cells in rheumatoid arthritis (RA).

**Methods:**

Serum and synovial fluid (SF) concentration levels of tryptase, chymase, and histamine were quantified using ELISA. After activating mast cells using IL-33, the production of TNF-α, IL-1β, IL-6, IL-17, RANKL, and MMPs was determined using real-time PCR and ELISA. Osteoclastogenesis was assessed in CD14+ monocytes from peripheral blood and SF, which were cultured with IL-33-activated mast cells, by counting TRAP-positive multinucleated cells.

**Results:**

The concentration levels of serum tryptase, chymase, and histamine and SF histamine were higher in patients with RA than in controls. FcεR1 and c-kit-positive mast cells were higher in RA synovium than in osteoarthritic (OA) synovium. Stimulation of mast cells by IL-33 increased the number of trypatse+chymase− and tryptase+chymase+ mast cells. IL-33 stimulation also increased the gene expression levels of TNF-α, IL-1β, IL-6, IL-17, RANKL, and MMP-9 in mast cells. Furthermore, IL-33 stimulated human CD14+ monocytes to differentiate into TRAP+ multinucleated osteoclasts. When CD14+ monocytes were co-cultured with mast cells, osteoclast differentiation was increased. Additionally, IL-33-activated mast cells stimulated osteoclast differentiation. The inhibition of intercellular contact between mast cells and monocytes using inserts reduced osteoclast differentiation.

**Conclusions:**

IL-33 increased inflammatory and tissue-destructive cytokines by activation of mast cells. Mast cells stimulated osteoclast differentiation in monocytes. Mast cells could stimulate osteoclastogenesis indirectly through production of tissue-destructive cytokines and directly through stimulation of osteoclast precursors.

## Background

Rheumatoid arthritis (RA) is characterized by intercellular interactions between macrophages, lymphocytes, synovial fibroblasts, osteoclasts, and chondrocytes, resulting in chronic inflammation and joint destruction [1]. Although the roles of most of these cells in RA pathogenesis are well-studied, mast cells and basophils remain important exceptions [2].

Mast cells, which are typically found in allergic diseases, are also found in RA synovial tissues and synovial fluid (SF). They are also known to be involved in RA pathogenesis. In RA, the synovium shows hyperplasia of mast cells, which can comprise up to 5% of all RA synovial cells. The number of mast cells in RA synovium is higher than that in osteoarthritic synovium [3]. Mast cells are mainly found around blood vessels, in the sublining area, and in areas of cartilage erosion. There are 2 types of mast cells: tryptase-positive MC_T_ cells and tryptase/chymase double-positive MC_TC_ cells [3]. MC_TC_ cells can be found in the normal synovium. The number of MC_T_ cells increases during early RA, indicating tissue inflammation, while that of MC_TC_ cells increases during advanced RA, indicating tissue remodeling [4].

The role of mast cells in RA is unclear. Some reports have demonstrated pathogenic inflammatory roles of mast cells in RA, while others have shown anti-inflammatory roles of mast cells in RA. Four broad lines of argument have been made for claiming that mast cells have an inflammatory role in RA. First, the number of mast cells increases in RA synovium and this correlates with disease activity parameters [3]. Patients with active RA have more mast cells in synovial tissues than patients with end-stage RA [5]. Second, mast cells release various inflammatory cytokines in RA synovial joints, including interleukin (IL)-6, tumor necrosis factor (TNF)-α, and IL-8. Third, histamine and other mast cell mediators have a potential for angiogenesis, fibroblast and macrophage activation, osteoclast differentiation, and leukocyte recruitment [6–10]. In our previous study, histamine, a preformed mediator of activated mast cells, was found to stimulate the production of receptor activator of nuclear factor kappa-Β ligand (RANKL) and osteoclast differentiation via the histamine H4 receptor [10]. Fourth, mast cell-deficient animal models are resistant to arthritis [11–13]. In contrast to these arguments, there is also evidence for the anti-inflammatory role of mast cells in RA. First, mast cells suppress monocyte and fibroblast activation, dendritic cell migration, and Th1 differentiation by inducing the secretion of IL-10, IL-4, IL-13, and transforming growth factor (TGF)-β [8, 14–16]. Second, the development of arthritis in animal models is not affected by the deficiency of mast cells [17, 18]. Third, serum tryptase levels in RA patients are negatively correlated with the levels of C-reactive protein [19].

In this study, we aimed to assess the inflammatory and tissue-destructive roles of mast cells in RA. We quantified the serum and SF concentration of mast cell mediators such as tryptase, chymase, and histamine in RA patients and characterized mast cell phenotypes of the mononuclear cells in the SF of RA patients. We also determined the expression and production of proinflammatory cytokines, IL-17, matrix metalloproteinases (MMPs), and RANKL after stimulating mast cells with IL-33. Finally, we examined the effects of activated mast cells on osteoclast differentiation in human monocytes.

## Methods

### Ethics statement and patients

Written informed consent was obtained from healthy volunteers and patients with RA and osteoarthritis. All RA patients fulfilled the 2010 ACR/EULAR classification criteria for RA [20]. Serum was collected from 20 RA patients and 20 healthy volunteers. Clinical data of RA patients is as follows: 12 females and 8 males, age 60 ± 16.8 years, rheumatoid factor (RF) 175.8 ± 49.1 IU/L, anti-CCP antibody 462.2 ± 163.2 IU/L, erythrocyte sedimentation rage (ESR) 59.6 ± 7.2 mm/h, C-reactive protein (CRP) 4.5 ± 1.0 mg/dl, DAS28-CRP 4.29 ± 0.2, and disease duration 2.8 ± 1.7 years. Thirteen patients were not taking medications, and others were taking oral disease-modifying antirheumatic drugs (DMARDs). SF was collected from 20 RA patients and 20 patients with osteoarthritis of knee joints. All SF samples were obtained through therapeutic arthrocentesis before steroid injection into the swollen joints. Clinical data of RA patients are as follows: 12 females and 8 males, age 53.9 ± 3.2 years, rheumatoid factor (RF) 143.5 ± 39.3 IU/L, anti-CCP antibody 418.1 ± 155.7 IU/L, ESR 51.5 ± 7.8 mm/h, CRP 3.7 ± 1.1 mg/dl, DAS28-CRP 3.8 ± 1.1, and disease duration 5.4 ± 1.6 years. Two patients were not taking medications, 1 patient was taking tofacitinib, and others were taking oral DMARDs. Synovial tissues were isolated from three RA patients (mean age 63.4 ± 4.6 years; range 38–76) and three osteoarthritis (OA) patients (mean age 63.5 ± 4.5 years; range 59–68) undergoing total knee-replacement surgery. Informed consent was obtained from all patients. All protocols were performed in accordance with relevant guidelines and regulations and were approved by the Institutional Review Board for Human Research in Konkuk University Hospital (KUH1010960).

### Reagents

Recombinant IL-33, RANKL, and M-CSF were purchased from R&D systems (Minneapolis, MN).

### Human mast cell line culture and stimulation

The human mast cell line (LUVA) was purchased from Kerafast (Kerafast, MA, USA) and maintained in StemPro-34 SFM at a concentration of 5 × 10^5^ cells/ml. The cells were grown at 37 °C with 5% CO_2_, and the medium was exchanged weekly with 50% fresh medium without exogenous cytokines. The mast cells were seeded in 6-well plates at a density of 5 × 10^4^ cells/ml. After 1 day, the medium was replaced with StemPro-34 SFM culture medium with or without cytokines. The mast cells were then stimulated with 100 ng/mL human recombinant IL-33 (R&D Systems, Inc.) for 24 and 72 h. The supernatants and RNA were harvested and stored at − 80 °C until analysis.

### Enzyme-linked immunosorbent assay (ELISA)

SF and serum samples obtained from healthy volunteers and patients with RA and osteoarthritis were subjected to tryptase, chymase, and histamine analyses using tryptase (Boster Biological Technology, CA), chymase (Aviva Systems Biology, CA), and histamine (Enzo Life Sciences Inc., NY) ELISA kits, according to the manufacturers’ instructions. The levels of cytokines such as TNF-α, IL-1, IL-6, IL-17, RANKL, MMP-9, and MMP-13 in the culture supernatants from human mast cells were measured using sandwich ELISA (R&D Systems), according to the manufacturer’s instructions. Absorbance at 405 nm was also measured using an ELISA microplate reader (Molecular Devices, CA).

### Flow cytometry

In the samples used for in vitro experiments, flow cytometry was performed after collecting the mast cells. Dead cells were excluded using the Fixable Viability Dye eFluor®506 (eBioscience). The cells were first stained with monoclonal antibodies. For surface staining, the cells were stained with mAbs against CD117 (c-kit)-APC (YB5.B8, IgG1κ) (eBioscience, CA). The cells were then washed, fixed, permeabilized, and stained with mAbs against FcεRI alpha-PE-Cy7 (AER-37-CRA1, IgG2b) (eBioscience), tryptase-FITC (G3, IgG1κ) (Santa Cruz Biotechnology, Inc., TX), and chymase-PE (CC1, IgG1κ) (Santa Cruz Biotechnology, Inc.) to detect intracellular cytokines. Appropriate isotype controls were used for gate setting. Cells were analyzed using a FACSCalibur flow cytometer and FlowJo software.

### Immunocytologic staining and confocal microscopy

To measure changes in protein expression, 4-μm-thick formalin-fixed and paraffin-embedded (FFPE) synovial sections were deparaffinized in xylene and rehydrated in ethanol and deionized water. Antigen retrieval was performed by heating these sections in a buffer solution (citrate buffer pH 6) (DAKO, Glostrup, Denmark) at 120 °C for 15 min. Slides were washed thrice (5 min each) with phosphate-buffered saline (PBS). The slides were then removed from PBS and each section was covered with 3% H_2_O_2_ solution (Sigma-Aldrich, Saint Louis, Missouri (MO)) for 15 min at room temperature to block endogenous peroxidase activity. After washing, non-specific binding was blocked by incubation in 10% normal goat serum in PBS for 30 min at room temperature. The blocking buffer was then removed and the sections were incubated overnight with c-kit (CD117) polyclonal antibody (DAKO), FcεR1 monoclonal antibody (Abcam, Cambridge, MA), FcεR1 polyclonal antibody (LSBio, Seattle, WA), FITC-conjugated tryptase antibody (Santa Cruz, California), and chymase monoclonal antibody (MBL, Nagoya, Japan) at 4 °C. The sections were then washed and incubated with the secondary antibodies, anti-rabbit IgG Alexa Fluor 594 and anti-mouse IgG-APC (Invitrogen, Carlsbad, California), for 2 h at room temperature. The nuclei were also stained with DAPI (Invitrogen). The slides were then covered with a fluorescent mounting medium (DAKO) and the stained sections were visualized under a Zeiss microscope (LSM 510 Meta; Carl Zeiss, Oberkochen, Germany) at × 200 and × 400 magnifications. The area of target+ cell from RA and OA synovium was measured in using ImageJ software.

### Real-time PCR for mRNA quantitation

mRNA was extracted from the samples using RNAzol B, according to the instructions of the manufacturer (Biotex, Friendswood, TX). The total mRNA (2 μg) was reverse transcribed at 42 °C using a SuperScript RT system (Takara). PCR was performed at a final volume of 20 μl in capillary tubes in a LightCycler (Roche Diagnostics). The reaction mixture contained 2 μl LightCycler FastStart DNAMaster Mix for SYBR Green I (Roche Diagnostics), 0.5 μM of each primer, 4 mM MgCl_2_, and 2 μl of the template DNA. All the capillaries were amplified in a LightCycler with the following program: activation of polymerase at 95 °C for 10 min, followed by 45 cycles of 10 s at 95 °C and 10 s at 60 °C (*beta-actin, IL-17, MMP-13*) or 57 °C (*TNF, IL-1β, IL-6, RANKL, MMP-9*), and a final 10 s at 72 °C. The temperature transition rate was 20 °C/s for all steps. Melting curve analyses were performed immediately after amplification, using the following program: 0 s (hold time) at 95 °C, 15 s at 71 °C, and 0 s (hold time) at 95 °C. The rate of temperature change was 20 °C/s for all steps except the final step, during which it was 0.1 °C/s. The melting peaks generated represented the quantity of each amplified product. The crossing point was defined as the maximum of the second derivative from the fluorescence curve. Negative controls, which contained all elements of the reaction mixture except for the template DNA, were also included. All samples were processed in duplicate.

### Isolation of peripheral blood (PB) monocytes and osteoclast differentiation

PB mononuclear cells were separated, washed thrice with sterile PBS, and resuspended in RPMI 1640 (Life Technologies, Grand Island, NY) supplemented with 10% fetal bovine serum (FBS), 2 mM L-glutamine, and 1% penicillin-streptomycin (henceforth called complete medium). Freshly isolated PBMCs were incubated at 37 °C in the complete medium and allowed to adhere for 45 min. The non-adherent cells were removed, and the adherent cells were washed with sterile PBS, harvested with a rubber policeman, and stained with monocyte-specific anti-CD14 monoclonal antibodies to assess the purity of the preparation. Ninety percent of the isolated cells were monocytes expressing CD14. Osteoclast precursors were prepared using the monocyte-enriched fraction from PB. The cells were co-cultured for 3 weeks in minimal essential medium (MEM)-α with 10% heat-inactivated FBS in the presence of 25 ng/ml rhM-CSF and IL-33/IL-33-stimulated mast cells. The medium was changed on day 3 and then on alternate days thereafter. On day 21, tartrate-resistant acid phosphatase (TRAP)-positive cells were identified using a leukocyte acid phosphatase kit, according to the manufacturer’s instructions (Sigma-Aldrich). The osteoclast precursors and mast cells/IL-33-stimulated mast cells were co-cultured in 24-well transwell plates separated by the membrane in lower and upper chambers, respectively (Costar, New York, NY). The medium was changed on day 3 and then every other day thereafter. On day 21, TRAP-positive cells were identified using a leukocyte acid phosphatase kit, according to the manufacturer’s protocol (Sigma-Aldrich).

### Western blotting

PB monocytes were incubated with or without IL-33 in the presence of RANKL. After incubation for 1 h, whole-cell lysates were prepared from approximately 1 × 10^7^ cells by homogenization in the lysis buffer and then centrifuged at 14,000 rpm for 15 min. Protein concentration in the supernatant was determined using the Bradford method (Bio-Rad, Hercules, CA). Protein samples were separated using 10% SDS–PAGE and transferred onto a nitrocellulose membrane (Amersham Pharmacia Biotech, Uppsala, Sweden). For western blotting, the membrane was pre-incubated with 0.5% skim milk in 0.1% Tween-20 and Tris-buffered saline (TTBS) at room temperature for 2 h. Primary antibodies against TRAF6, phospho-Src, Src, phospho-JNK, JNK, phospho-ERK, ERK, phospho-p38, p38, phospho-Akt, Akt, phospho-IκBα, IκBα, phospho-c-Jun, and c-Jun (Cell Signaling Technology Inc., Danvers, MA), diluted 1:1000 in 5% BSA-0.1% Tween-20/TBS, were added and incubated overnight at 4 °C. After washing the membrane 4 times with TTBS, horseradish peroxidase-conjugated secondary antibody was added and incubated for 1 h at room temperature. After TTBS washing, hybridized bands were detected using the ECL detection kit and Hyperfilm-ECL reagents (Amersham Pharmacia).

### Statistical analysis

All data are expressed as mean ± standard error of the mean (SEM). Statistical differences between two groups were assessed using Mann-Whitney *U* test and those between more than three groups were assessed using one-way analysis of variance (ANOVA) with Bonferroni’s multiple comparison post-hoc test. Differences with *p* < 0.05 were considered statistically significant.

## Results

### Serum and SF concentration levels of tryptase, chymase, and histamine in patients with RA

**Fig. 1:**
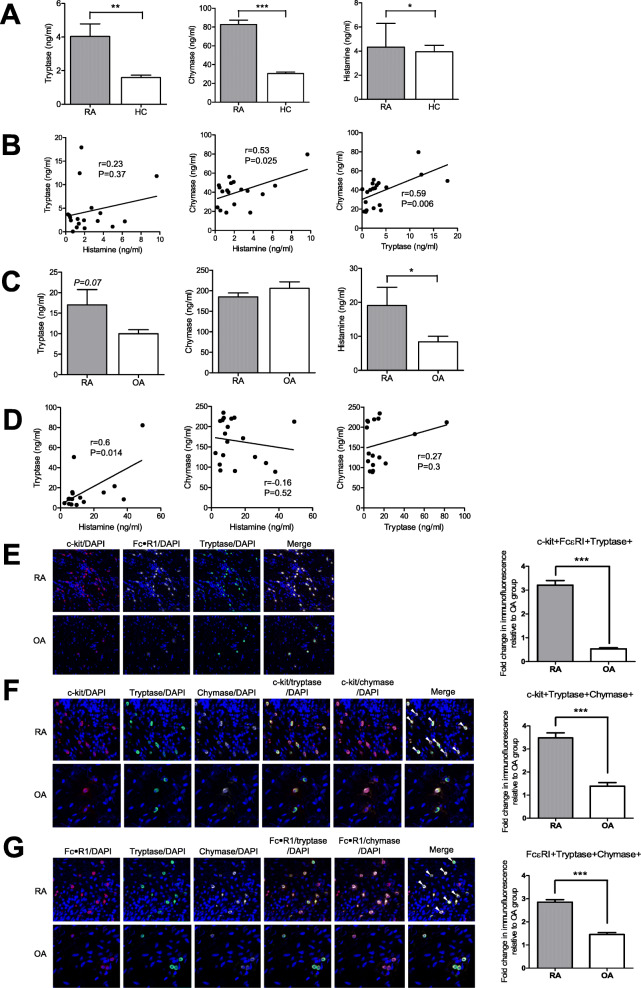
Serum and synovial fluid (SF) concentration levels of tryptase, chymase, and histamine and the expression of c-kit, FcεRI, tryptase, and chymase in the synovial tissues of rheumatoid arthritis (RA) patients. **a** The serum concentration of tryptase, chymase, and histamine in RA patients and healthy controls was measured using ELISA. **b** The associations between serum concentration levels of tryptase, chymase, and histamine in RA patients were analyzed using Pearson’s correlation test. **c** The SF concentration of tryptase, chymase, and histamine in patients with RA and osteoarthritis was measured using ELISA. **d** The associations between SF concentration levels of tryptase, chymase, and histamine in RA patients were analyzed using Pearson’s correlation test. Data are presented as mean ± SEM. *p < 0.05, **p < 0.01, and ***p < 0.001. **e** Synovial tissues of patients with RA and osteoarthritis (OA) were labeled with anti-c-kit (red), anti-FcεRI (white), and anti-tryptase (green) antibodies and photographed under appropriate filters (original magnification = × 200, scale bar = 50 μm). **f** Synovial tissues of patients with RA and OA were labeled with anti-c-kit (red), anti-tryptase (green), and anti-chymase (white) antibodies and photographed under appropriate filters (original magnification = × 400, scale bar = 20 μm). **g** Synovial tissues of patients with RA and OA were labeled with anti-FcεRI (red), anti-tryptase (green), and anti-chymase (white) antibodies and photographed under appropriate filters (original magnification = × 400, scale bar = 20 μm). Merged image shows co-localization of the three markers (yellow). Sections were counterstained with DAPI (blue). Figures are representatives of three independent experiments. RA, rheumatoid arthritis; HC, healthy control; OA, osteoarthritis

The serum concentration levels of tryptase, chymase, and histamine were measured using ELISA in 20 patients with RA and 20 healthy controls. The clinical characteristics of the RA patients were as follows: age 60 ± 16.8 years, rheumatoid factor (RF) 175.8 ± 49.1 IU/L, anti-CCP antibody 462.2 ± 163.2 IU/L, erythrocyte sedimentation rage (ESR) 59.6 ± 7.2 mm/h, C-reactive protein (CRP) 4.5 ± 1.0 mg/dl, DAS28-CRP 4.29 ± 0.2, and disease duration 2.8 ± 1.7 years. The serum concentration of tryptase was significantly higher (*p* = 0.002) in RA patients (4.04 ± 0.74 ng/ml) than in healthy controls (1.59 ± 0.14 ng/ml). The serum concentration of chymase was also significantly higher (*p* < 0.0001) in RA patients (82.77 ± 4.6 ng/ml) than in healthy controls (30.54 ± 1.58 ng/ml). Similarly, the serum concentration of histamine was also significantly higher (*p* = 0.048) in RA patients (4.33 ± 1.67 ng/ml) than in healthy controls (3.94 ± 0.54 ng/ml) (Fig. 1a). In RA patients, the serum concentration levels of chymase and histamine were correlated (*r* = 0.53, *p* = 0.025), as were those of chymase and tryptase (*r* = 0.59, *p* = 0.006). However, there was no correlation between the serum concentration levels of tryptase and histamine (*r* = 0.23, *p* = 0.37) (Fig. 1b).

The SF concentration of tryptase tended to be higher in RA patients (17.03 ± 3.73 ng/ml) than in patients with osteoarthritis (9.99 ± 0.97 ng/ml), although this difference was not statistically significant (*p* = 0.07). However, the SF concentration of histamine was significantly higher (*p* = 0.019) in RA patients (19.1 ± 5.33 ng/ml) than in patients with osteoarthritis (8.38 ± 1.65 ng/ml) (Fig. 1c). In RA patients, the SF concentration of tryptase was correlated with that of histamine (*r* = 0.6, *p* = 0.014). However, the SF concentration levels of chymase and histamine were not correlated (*r* = − 0.15, *p* = 0.52), similar to those of chymase and tryptase (*r* = 0.27, *p* = 0.3) (Fig. 1d). The c-kit-, FcεRI-, tryptase-, and chymase-positive cells in RA and OA synovial tissues were counted using multiple-fluorescence staining and confocal microscopy. C-kit, FcεRI, and tryptase triple-positive cells were abundant in the RA synovium, but negligible in the OA synovium (Fig. 1e). By triple immunofluorescent labeling for c-kit, tryptase, and chymase, c-kit expression was found to consistently overlap with the expression of tryptase and chymase (Fig. 1f). FcεRI expression also overlapped with the expression of tryptase and chymase (Fig. 1g).

### Characterization of mast cells in RA SF

SFMCs were isolated from 6 patients with RA. We quantified c-kit-, FcεRI-, tryptase-, and chymase-positive cells among these SFMCs. C-kit positive cells comprised 7.33 ± 1.81%, while FcεRI-positive cells comprised 20.23 ± 5.42%, of the total SFMCs. C-kit and FcεRI double-positive cells comprised 10.46 ± 3.26% of the total SFMC. Tryptase- and chymase-positive cells comprised 29.82 ± 8.06% and 29.45 ± 5.19% of total SFMC, respectively. Among c-kit and FcεRI double-positive cells, there was no difference in the proportions of tryptase- and chymase-positive cells. Further, the characteristics of SFMCs from RA and osteoarthritis patients were not different (supplementary Figure 1).

### Stimulatory effects of IL-33 on mast cell lines (LUVA) phenotypes

**Fig. 2:**
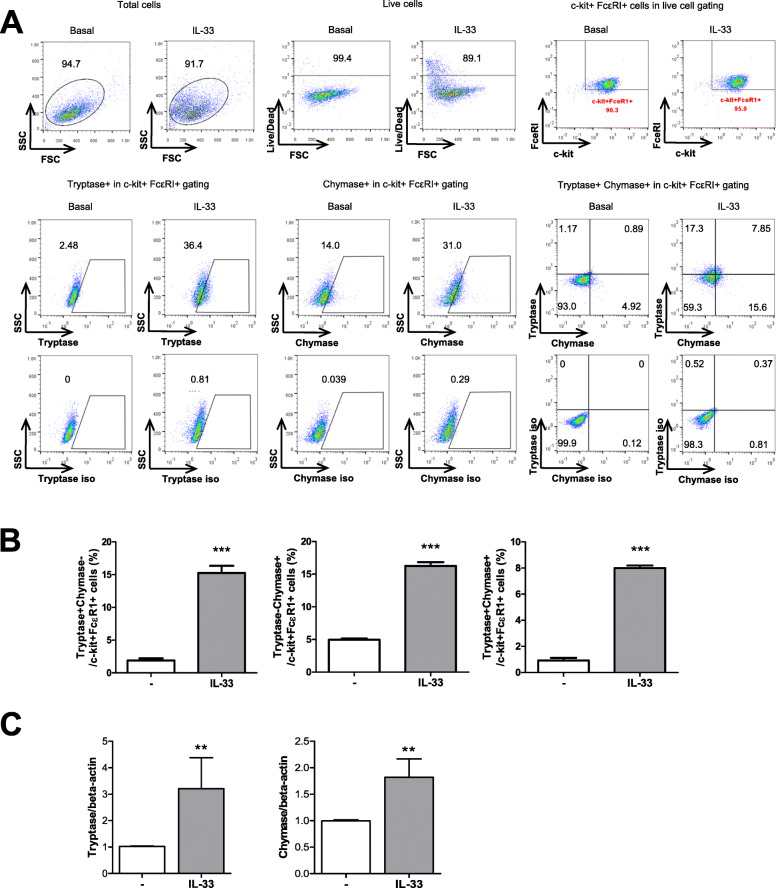
Effect of IL-33 on the expression of tryptase and chymase in mast cells. **a** Human mast cells were culture with 100 ng/ml of IL-33 for 24 h, after which c-kit+ FcεRI+ cells were gated for further analysis. **b** Percentages of tryptase- and chymase-positive mast cells were measured by flow cytometry. **c** Gene expression levels of tryptase and chymase were measured using real-time PCR. Data were normalized to the expression level of beta-actin and reported in relative expression units. Data are presented as mean ± SEM from four independent experiments. *p < 0.05 and **p < 0.01

IL-33 is major stimulatory cytokine for mast cell lines (LUVA). After stimulation with IL-33, we observed an increase in trypatse+chymase− and tryptase+chymase+ mast cells. Tryptase−chymase+ mast cells also increased after IL-33 stimulation, although this increase was not statistically significant (Fig. 2a, b). The gene expression levels of both tryptase and chymase in the mast cells also increased after IL-33 stimulation (Fig. 2c). We have added the gating strategy in the supplementary figure (supplementary Figure 2).

### Effects of IL-33 on the gene expression and production of proinflammatory and tissue-destructive cytokines in mast cell lines (LUVA)

**Fig. 3:**
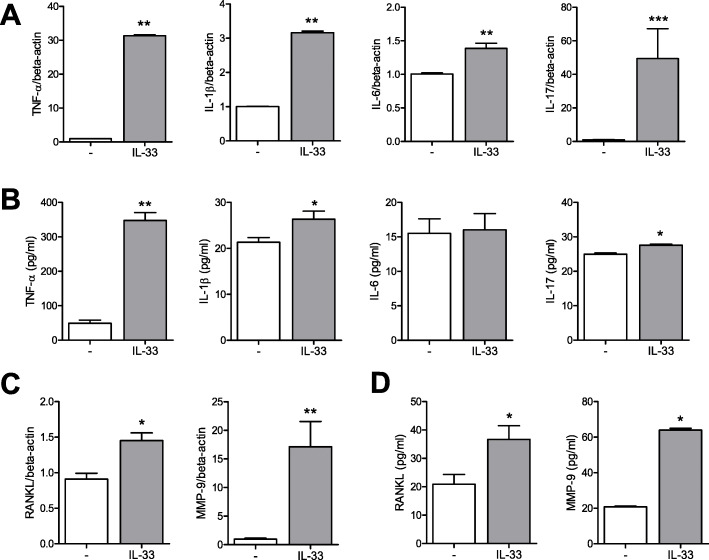
Gene expression levels and production of proinflammatory cytokines and tissue-destructive molecules in mast cells stimulated by IL-33. **a** After mast cells were cultured with 100 ng/ml of IL-33, the gene expression levels of TNF-α, IL-1β, IL-6, and IL-17 were determined by real-time PCR. **b** Production of TNF-α, IL-1β, IL-6, and IL-17 in the culture media was quantified using ELISA. **c** After mast cells were cultured with 100 ng/ml of IL-33 for 24 h, the gene expression levels of RANKL and MMP-9 were determined by real-time PCR. **d** Production of RANKL and MMP-9 in the culture media was quantified using ELISA. Data from real-time PCR analyses were normalized to the expression level of beta-actin and reported in relative expression units. Data are presented as mean ± SEM from six independent experiments. *p < 0.05, **p < 0.01, and ***p < 0.001

When mast cells were stimulated by IL-33, the gene expression levels of proinflammatory cytokines such as TNF-α, IL-1β, IL-6, and IL-17 increased (Fig. 3a). IL-33 stimulation also increased the production of TNF-α, IL-1β, and IL-17 in the culture media (Fig. 3b). IL-33 also affected bone and cartilage destruction by stimulating the gene expression and production of RANKL and MMP-9 in mast cells (Fig. 3c, d). However, IL-33 did not affect the production of MMP-13 (data not shown).

### Effects of IL-33 on osteoclast differentiation in PB and SF monocytes

**Fig. 4:**
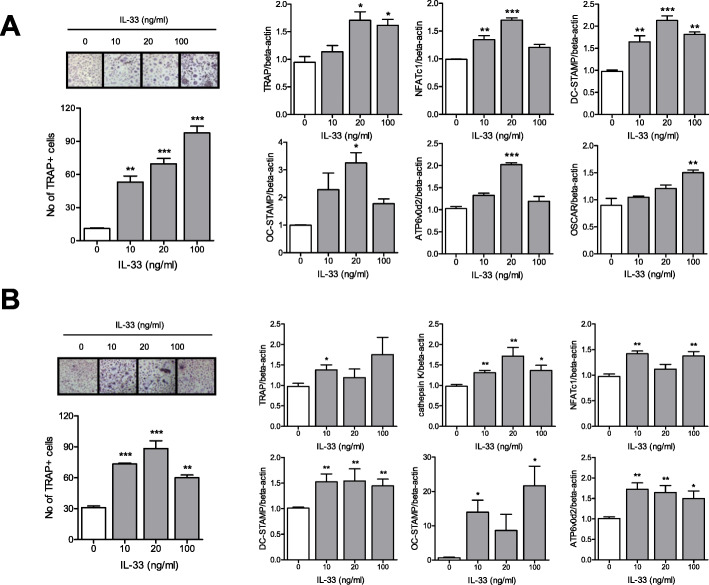
Effect of IL-33 on osteoclast differentiation in peripheral blood (PB) and synovial fluid (SF) monocytes. **a** PB and **b** SF CD14+ monocytes were cultured with various doses of IL-33 along with 25 ng/ml M-CSF. After 21 days of culturing, TRAP-positive multinucleated cells, mature osteoclasts, were counted. Figures represent data from one of three independent experiments, and the bars represent mean ± SEM. Gene expression levels of osteoclast markers such as TRAP, NFATc1, DC-STAMP, OC-STAMP, and ATP6v0d2 were measured using real-time PCR. Data were normalized to the expression level of beta-actin and reported in relative expression units. *p < 0.05, **p < 0.01, and ***p < 0.001

The effect of IL-33 on osteoclast differentiation depends on the type of osteoclast progenitors involved. IL-33 has anti-osteoclastogenic functions in human cord blood progenitors, mouse cell lines such as RAW264.7, and bone marrow cells. However, it stimulates osteoclast differentiation in human CD14+ monocytes [21–24]. Here, we attempted to define the effect of IL-33 on osteoclast differentiation in human PB CD14+ monocytes. IL-33 stimulated the differentiation of CD14+ monocytes to TRAP+ multinucleated osteoclasts in a dose-dependent manner. The gene expression levels of TRAP, NFATc1, DC-STAMP, OC-STAMP, ATP6v0d2, and OSCAR in PB CD14+ monocytes increased upon IL-33 stimulation (Fig. 4a). Similar to PB monocytes, the gene expression levels of TRAP, NFATc1, DC-STAMP, OC-STAMP, ATP6v0d2, and OSCAR also increased in SF monocytes upon IL-33 stimulation (Fig. 4b).

### Effects of IL-33 on RANKL-induced osteoclast differentiation in PB and SF monocytes

**Fig. 5:**
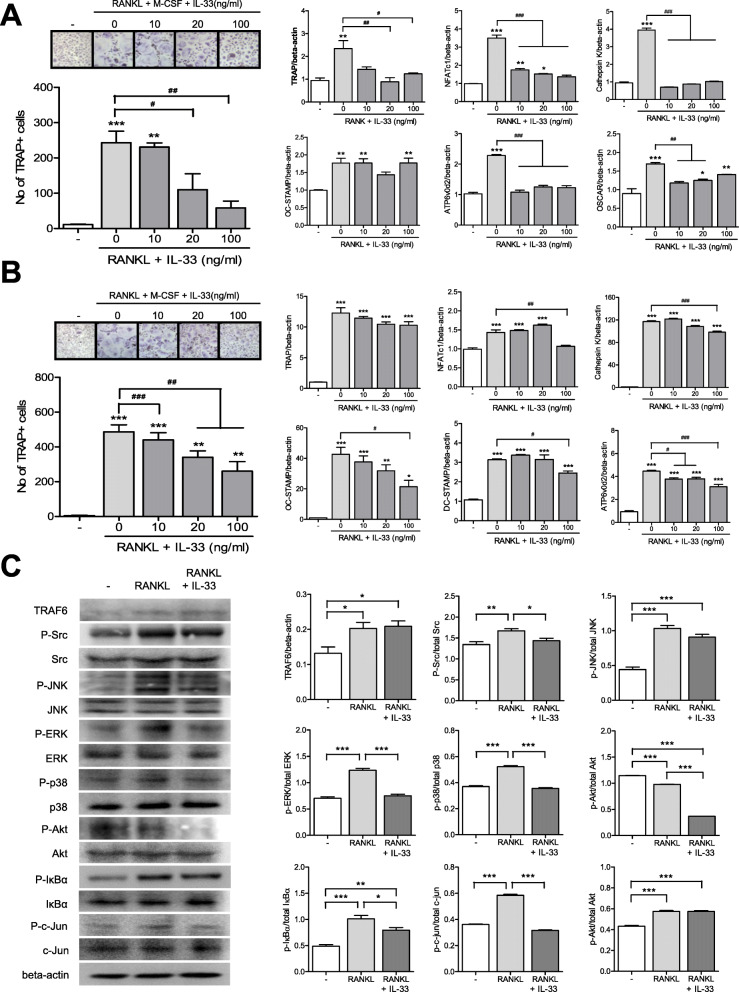
Effects of IL-33 on RANKL-induced osteoclast differentiation in peripheral blood (PB) and synovial fluid (SF) monocytes. **a** PB and **b** SF CD14+ monocytes were cultured with various doses of IL-33 in the presence of 25 ng/ml M-CSF and 10 ng/ml RANKL. After 21 days of culturing, TRAP-positive multinucleated cells, mature osteoclasts, were counted. Figures represent data from one of three independent experiments, and the bars represent mean ± SEM. Gene expression levels of osteoclast markers such as TRAP, NFATc1, DC-STAMP, OC-STAMP, and ATP6v0d2 were measured using real-time PCR. Data were normalized to the expression level of beta-actin and reported in relative expression units. *p < 0.05, **p < 0.01, and ***p < 0.001 indicate significant difference from the value for nil condition and ^##^*p* < 0.01 indicates significant difference between two conditions. **c** Immunoblotting was performed for TRAF6, phospho-Src, Src, phospho-JNK, JNK, phospho-ERK, ERK, phospho-p38, p38, phospho-Akt, Akt, phospho-IκBα, IκBα, phospho-c-Jun, c-Jun, and beta-actin in PB CD14+ monocytes treated with IL-33 and untreated controls in the presence of 10 ng/ml RANKL for 1 h. Data were normalized to the expression level of beta-actin and reported in relative expression units. Bars show mean ± SEM from 3 independent experiments. *p < 0.05, **p < 0.01, and ***p < 0.001

RANKL is a major stimulant for the differentiation of mature osteoclasts. When we cultured PB monocytes with RANKL and M-CSF, TRAP+ multinucleated osteoclasts were differentiated. However, IL-33 reduced this RANKL-induced osteoclast differentiation. The gene expression levels of TRAP, NFATc1, cathepsin-K, ATP6v0d2, and OSCAR in PB monocytes were increased upon RANKL stimulation but decreased after the addition of IL-33 (Fig. 5a). Further, RANKL and IL-33 treatment had similar effects on osteoclast differentiation in SF monocytes as on PB monocytes (Fig. 5b).

As described in the previous sections, although IL-33 stimulated osteoclastogenesis, when IL-33 was added in the culture system with RANKL, osteoclastogenesis was reduced. To determine the change in intracellular signaling caused by IL-33 in RANKL-induced osteoclastogenesis, PB monocytes were cultured with or without IL-33 in the presence of RANKL. IL-33 decreased RANKL-induced phosphorylation of Src, ERK, p38 MAPK, IκBα, and c-jun. However, the expression of TRAF6 and the phosphorylation of JNK and Akt were not affected by IL-33 (Fig. 5c).

### Effects of mast cells on osteoclast differentiation in PB monocytes

**Fig. 6:**
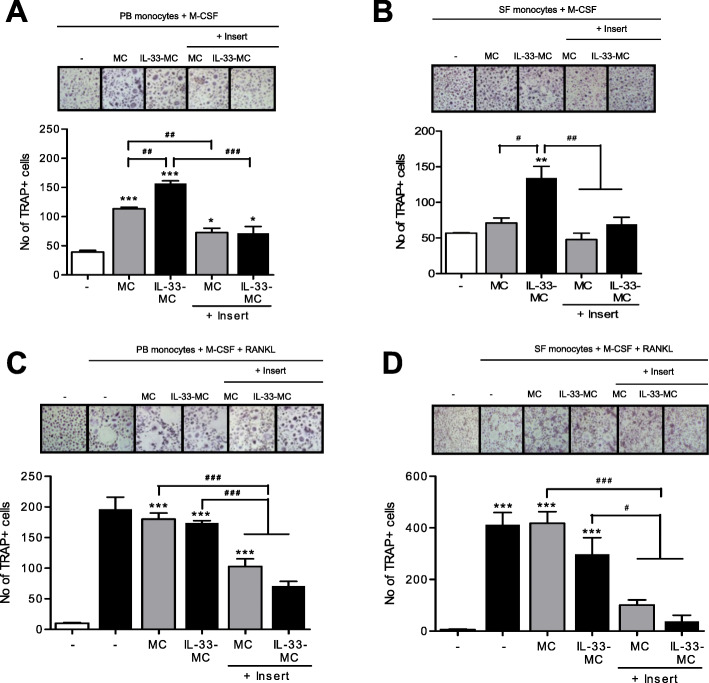
Effects of mast cells on osteoclast differentiation in peripheral blood (PB) and synovial fluid (SF) monocytes. **a** PB and **b** SF CD14+ monocytes were co-cultured with mast cells or IL-33-simulated mast cells in the presence of M-CSF for 21 days, after which TRAP-positive multinucleated cells, mature osteoclasts, were counted. To determine the effect of cell-cell contact, transwell inserts were placed between the mast cells and the monocytes. **c** PB and **d** SF CD14+ monocytes were co-cultured with mast cells or IL-33-simulated mast cells in the presence of M-CSF and RANKL for 21 days, after which TRAP-positive multinucleated cells, mature osteoclasts, were counted. Figures represent one of 3 independent experiments, and the bars represent mean ± SEM. *p < 0.05, **p < 0.01, and ***p < 0.001 indicate significant difference from the value for nil condition, while ^#^*p* < 0.05, ^##^*p* < 0.01, and ^###^*p* < 0.001 indicate significant difference between two conditions

Co-culturing PB CD14+ monocytes with mast cells increased osteoclast differentiation regardless of the activation of mast cells. However, IL-33-activated mast cells stimulated more osteoclast differentiation. To determine the osteoclastogenic effect of monocyte-mast cell contact, transwell inserts were placed between monocytes and mast cells in the co-culture system. When intercellular contact was inhibited by the transwell insert, osteoclast differentiation decreased (Fig. 6a). When SF monocytes were co-cultured with mast cells, IL-33-activated mast cells induced more osteoclast differentiation than non-activated mast cells. However, the transwell inserts reduced osteoclast differentiation by IL-33-activated mast cells (Fig. 6b). When RANKL was added in the co-culture system, osteoclast differentiation was induced, regardless of co-culture with mast cells. The osteoclastogenic effect of IL-33-activated mast cells was not different from that of non-activated mast cells. However, the transwell inserts again reduced osteoclast differentiation in both activated and non-activated mast cells (Fig. 6c). The effect of transwell inserts on osteoclast differentiation in the presence of RANKL was similar in SF and PB monocytes (Fig. 6d).

## Discussion

Mast cells have so far not been a major focus of RA research; results from in vitro cellular experiments and in vivo animal studies have been inconsistent. Synovial mast cells have been shown to be associated with inflammation, autoantibody production, and high disease activity in early RA [25]. In this study, we investigated the role of mast cells in bone destruction during RA.

We quantified the serum and SF concentration levels of mast cell mediators, including tryptase, chymase, and histamine. The serum concentration levels of all three were higher in RA patients than in patients with osteoarthritis. Further, the concentration of chymase was correlated with that of tryptase and histamine. Although their concentration levels were higher in SF than in serum samples, only histamine was significantly higher in RA than in osteoarthritis. Among the important mast cell mediators, tryptase has been shown to be of clinical and experimental significance in RA. Serum tryptase is correlated with RA disease activity indices, and anti-tryptase antibodies have been detected in RA synovial tissues and sera. Further, tryptase inhibits apoptosis in RA synovial fibroblasts, while tryptase inhibition reduces inflammatory parameters in animal models of arthritis [26–28]. In contrast, the clinical and pathological roles of chymase in RA remain unclear. As for histamine, its production has been shown to be increased in RA, although histamine levels do not reflect clinical disease activity [10, 29].

Previous studies have shown that the number of mast cells increases in RA synovial tissue, especially in early RA [25, 30]. However, the proportion of mast cells in the SF has not been investigated so far. We found that c-kit and FcεRI double-positive mast cells comprised over 10% of SFMCs in RA patients, suggesting that a considerable number of mast cells exists in the SF, which may play a major role in inflammation and tissue destruction. We also characterized synovial mast cells using confocal staining and flow cytometric analyses. Compared with osteoarthritic synovial tissues, RA synovial tissues had more c-kit- and FcεRI-positive mast cells, which produced both tryptase and chymase. Tryptase and chymase are markers for mast cell activation, and as mast cells in the RA synovium had both tryptase and chymase, it is likely that these mast cells are activated.

Mast cells are activated upon stimulation by cytokines such as IL-3, IL-4, IL-5, and IL-33, immune complexes, toll-like receptor-2 and 4, Fcγ receptors, and direct interaction with helper T-cells [31–34]. IL-33, a member of the IL-1 family, which is expressed in epithelial cells, fibroblasts, dendritic cells, and macrophages [35], is also associated with RA. Its level is increased in the serum and SF of RA patients and decreases with RA treatment [35]. IL-33 stimulates mast cells to produce TNF and IL-6, two major proinflammatory cytokines, in RA [36].

Here, we used IL-33 to activate mast cells, and analyzed subsequent changes in mast cell phenotype and expression of proinflammatory cytokines and tissue-degrading molecules such as RANKL and MMPs. IL-33 stimulation increased the number of tryptase-positive and tryptase/chymase-positive mast cells, suggesting that IL-33 was effective in stimulating mast cells and that tryptase is the best marker for mast cell activation. A previous study showed that synovial fibroblast-derived IL-33 promotes the expression of tryptase in mast cells [37], indicating that synovial fibroblasts may be a major source of IL-33 for the activation of mast cells in the RA synovium. IL-33 also increased the expression of TNF-α, IL-1β, IL-6, and IL-17, suggesting that activated mast cells may be a major source of proinflammatory cytokines in the RA synovium. Moreover, IL-33 stimulated the expression of RANKL and MMP-9, which are associated with bone and cartilage destruction in RA. This result also suggests that activated mast cells may have joint tissue-destructive roles in RA. Although RANKL is crucial for osteoclast differentiation, osteoclastogenesis occurs in the joints of RA patients without RANKL. Instead, TNF-α, IL-1β, and Th17 cytokines induce osteoclast differentiation from their precursors [38–40]. The stimulatory effect of IL-33 on RANKL production in monocytes suggests that IL-33 could have a bone-destructive role in RA.

IL-33 induces osteoclast differentiation in human PB and SF monocytes through its receptor ST2 and has a synergistic effect on osteoclast differentiation with RANKL [23, 41]. However, IL-33 has a double-sided effect on osteoclast differentiation, and this is not yet well defined. We studied the role of IL-33 on osteoclast differentiation in three different osteoclast culture systems. In addition to PB monocytes, we also used SF monocytes from RA patients as osteoclast precursors. First, we stimulated osteoclast precursors with IL-33 in the absence of RANKL. IL-33 induced osteoclast differentiation in both PB and SF monocytes, indicating the osteoclastic effect of IL-33 independent of RANKL stimulation. This result is consistent with those of a previous study on human CD14+ monocytes [23]. Second, we added IL-33 in the osteoclast culture systems in the presence of RANKL and found that IL-33 reduced RANKL-induced osteoclastogenesis. This result is in contrast to the findings from the previous study [23]. We also found that the phosphorylation of signal molecules plays a crucial role in RANKL-induced osteoclastogenesis. IL-33 inhibited the phosphorylation of src, ERK, p38, NF-κB, and c-jun. Thus, IL-33 stimulated osteoclastogenesis, but reduced the osteoclastogenic effect of RANKL. Future studies should aim to explore the underlying mechanisms of the inhibitory effect of IL-33 on RANKL-induced osteoclastogenesis in more detail.

Finally, we investigated the effect of intercellular contact between mast cells and osteoclast precursors. Co-culturing mast cells with osteoclast precursors increased osteoclast differentiation. IL-33-stimulated mast cells induced more osteoclast differentiation than non-activated mast cells. To determine the effect of intercellular contact between mast cells and osteoclast precursors, we inhibited their contact using transwell inserts. The inhibition of cellular contact decreased osteoclast differentiation, suggesting that cell-cell contact is the major mechanism of mast cell-induced osteoclast differentiation, rather than cytokine stimulation. The addition of RANKL to the culture system masked the effects of IL-33-activated mast cells, indicating that RANKL has a stronger osteoclastogenic effect than IL-33. However, the inhibition of contact between mast cells and osteoclast precursors diminished the osteoclastogenic effect of RANKL.

## Conclusions

Mast cells and their mediators such as tryptase, chymase, and histamine were increased in the synovial tissues and SF of RA patients, compared to healthy controls and osteoarthritis patients. Mast cells stimulated osteoclast differentiation through intercellular contact. The inhibition of mast cells could therefore be a promising new therapeutic strategy for the prevention of joint destruction in RA.

## Supplementary Information

**Additional file 1: Supplementary Figure 1.** Characterization of mast cells in RA SF. (A) The proportion of mast cell marker positive cells in RA SFMC. (B) The proportion of chymase and/or tryptase positive cells in c-kit positive and FcεR1 positive mast cells of RA SF. (C) The proportion of mast cell in synovial fluid in patients with RA compared with OA.

**Additional file 2: Supplementary Figure 2.** Gating strategies to determine tryptase+ and chymase+ cell populations in the Mast cell lines (LUVA). The flow cytometer experiment results were analyzed as follows. All populations can then be analyzed for further markers, such as SSC and FSC, which are size and granularity of the cell. Live populations can then be analyzed for further markers, such as Fixable Viability Dye eFluor®506 (eBioscience), which are live/dead markers. Even further, the live cells can be analyzed further for expression of c-kit and FcεRI, which are mast cell markers. These mast cells populations can the be analyzed for further markers, such as tryptase and chymase.

## Data Availability

The datasets generated and/or analyzed in this study are available from the corresponding author upon reasonable request.

## Abbreviations

RA: Rheumatoid arthritis
OA: Osteoarthritis
SF: Synovial fluid
MC: Mast cells
TNF: Tumor necrosis factor
IL: Interleukin
RANKL: Receptor activator of nuclear factor κB ligand
MMPs: Matrix metallopeptidases
TGF: Transforming growth factor
CD: Cluster of differentiation
TRAP: Tartrate-resistant acid phosphatase
PBMC: Peripheral blood mononuclear cell
ELISA: Enzyme-linked immunosorbent assay
PCR: Polymerase chain reaction
SDS–PAGE: Sodium dodecyl sulfate–polyacrylamide electrophoresis
SEM: Standard error mean
Th: Helper T cell
TTBS: Tween 20 in Tris-buffered saline
