# Supplementary figures and images for: Regulation of osteoclastogenesis by mast cell in rheumatoid arthritis
Source: Arthritis Res Ther. 2021 Apr 21;23:124. doi: 10.1186/s13075-021-02491-1 (PMC8059019; doi:10.1186/s13075-021-02491-1)

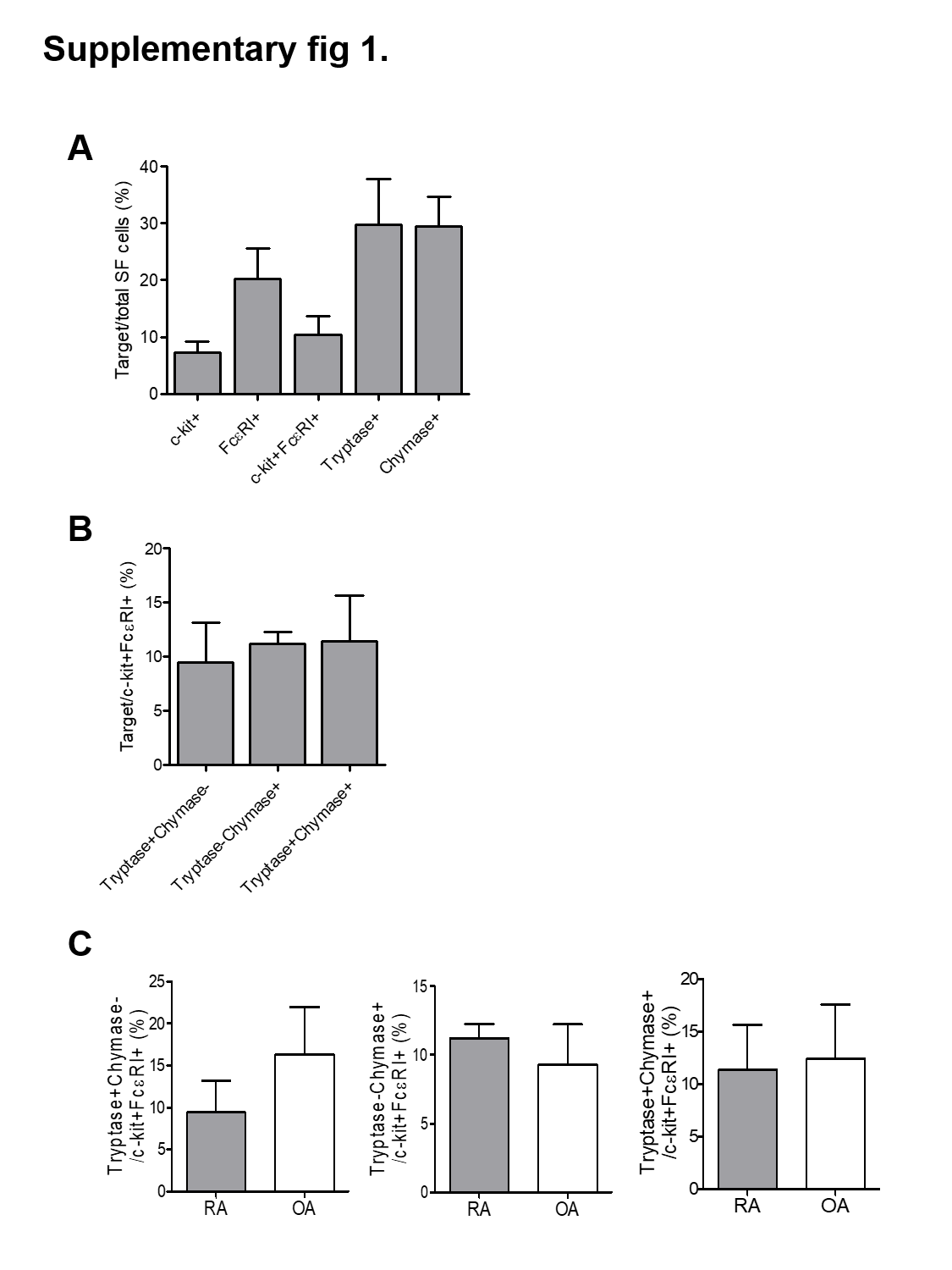

Supplement: Supplementary file 1 — Additional file 1: Supplementary Figure 1. Characterization of mast cells in RA SF. (A) The proportion of mast cell marker positive cells in RA SFMC. (B) The proportion of chymase and/or tryptase positive cells in c-kit positive and FcεR1 positive mast cells of RA SF. (C) The proportion of mast cell in synovial fluid in patients with RA compared with OA. [file 13075_2021_2491_MOESM1_ESM.tif]

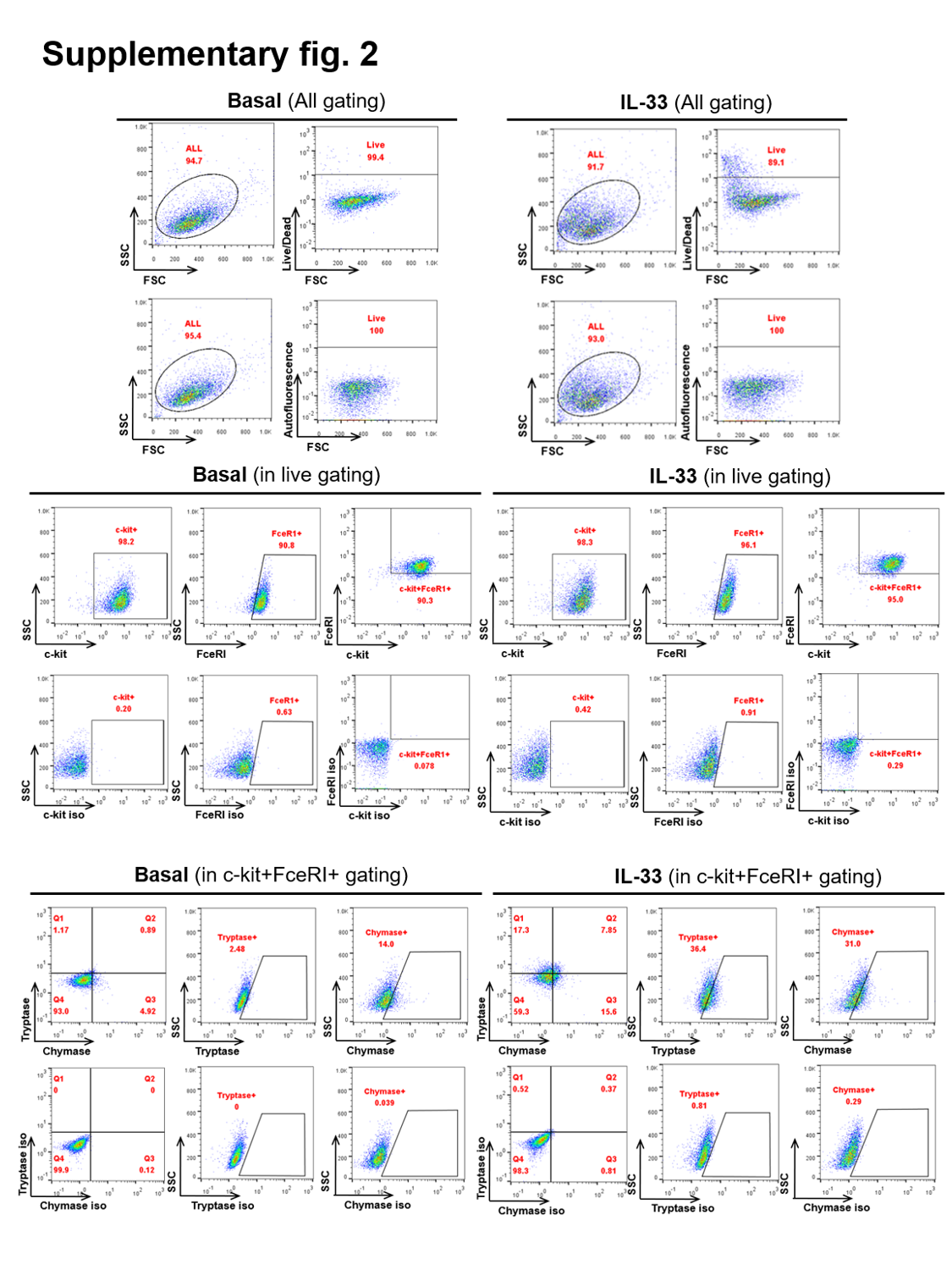

Supplement: Supplementary file 2 — Additional file 2: Supplementary Figure 2. Gating strategies to determine tryptase+ and chymase+ cell populations in the Mast cell lines (LUVA). The flow cytometer experiment results were analyzed as follows. All populations can then be analyzed for further markers, such as SSC and FSC, which are size and granularity of the cell. Live populations can then be analyzed for further markers, such as Fixable Viability Dye eFluor®506 (eBioscience), which are live/dead markers. Even further, the live cells can be analyzed further for expression of c-kit and FcεRI, which are mast cell markers. These mast cells populations can the be analyzed for further markers, such as tryptase and chymase. [file 13075_2021_2491_MOESM2_ESM.tif]
